# The Association between Non-Medical Prescription Drug Use and Suicidal Behavior among United States Adolescents

**DOI:** 10.3934/publichealth.2014.4.226

**Published:** 2014-11-04

**Authors:** Amanda L. Divin, Keith J. Zullig

**Affiliations:** 1 Department of Health Sciences and Social Work, Western Illinois University, Macomb, IL 61455, USA; 2 Department of Social and Behavioral Sciences, West Virginia University, Morgantown, WV 26501, USA

**Keywords:** prescription drug use, adolescents, suicide, drug use, suicidal ideation

## Abstract

Adolescence represents a vulnerable time for the development of both drug use/abuse and mental illness. Although previous research has substantiated a relationship between drug use and suicidal behavior, little research has examined this relationship with non-medical prescription drug use. Given the growing prevalence of non-medical prescription drug use (NMPDU) among adolescents, this study explored the association between NMPDU and suicidal behavior. Nationally representative data were derived from 16, 410 adolescents who completed the 2009 National Youth Risk Behavior Survey. Approximately 19.8% of participants reported lifetime NMPDU. NMPDU was associated with significantly increased odds of suicidal behavior (*P* < 0.01), with seriously considering attempting suicide and making a plan about attempting suicide representing the strongest correlates for males and females. Results suggest the importance of 1) continued reinforcement of drug education programs in high school begun at earlier ages and 2) mental health care and screenings among adolescents.

## 1. Introduction

Nonmedical prescription drug use (NMPDU) has reached epidemic proportions in the United States (US). Currently, it ranks second only to marijuana as the most prevalent drug problem in the US [1]. Frequently defined as intentional use of a prescription medication without a valid doctor's prescription [2],[3], NMPDU has been on the rise since the early 1990s [4], and appears to be disproportionately affecting adolescents. For example, according to the 2012 Partnership Attitude Tracking Study (PATS), NMPDU in adolescents has increased 33% since 2008, with one in four adolescents reporting NMPDU in their lifetime [5].

NMPDU among adolescents is associated with significant morbidity and mortality. According to the 2009 Drug Abuse Warning Network (DAWN), there were approximately 66,000 emergency department visits due to nonmedical use of pharmaceuticals among 12–17 year olds [6], with 95.4% of visits for drug-related suicide attempts involving pharmaceuticals [7]. Moreover, unintentional poisoning, partially driven by prescription drug overdose, was the second leading cause of accidental death among 15–19 year olds in the US [8], while suicide was the overall third leading cause of death in this group [9].

NMPDU has also been associated with a variety of psychopathologies including depression [6],[10]–[14] and suicidal behavior [10],[15],[16]. Although NMPDU, depression, and suicidal behavior (e.g., suicide ideation, plans, and attempts) occur frequently during adolescence, little research has examined the relationship these variables may share among adolescents. What is known, however, is that among adolescents, both prescription medication use [17],[18] and suicide [19] increase with age. In addition, females are more likely to use prescription medications [18], suffer from depression [20],[21], and attempt suicide [22],[23]. Nevertheless, the comorbidity of substance use and psychiatric disorders is considered the norm, not the exception [24], and adolescents who suffer from depression and engage in drug use are significantly more likely to attempt suicide [25].

While depression is one of the most treatable psychiatric illnesses [26], many adolescents may ignore or not recognize the symptoms, instead relying on drug use to self-medicate [27]. Thus, self-treatment of such psychological distress may be one of the factors which contributes to the increasing prevalence in substance use. Individuals with higher levels of depressive symptoms comprise a special group of nonmedical prescription drug users at higher risk for other psychopathology [28]. Given the propensity for both depression [29] and experimentation with substance use [30] to begin early and increase with age, as well as the known comorbidity of depression and substance use in adolescence [31],[32] more research exploring the relationship between NMPDU, depression, and suicidal behavior in adolescents is necessary. The purpose of this study was to further explore the relationship between NMPDU, depression, and suicidal behavior in a large, nationally representative sample of adolescents. It was hypothesized that NMPDU would be related to depression; and suicide ideation, plans, and attempts.

## 2. Materials and Method

### 2.1. Sampling Procedure

The 2009 US Centers for Disease Control and Prevention's (CDC) National Youth Risk Behavior Surveillance (YRBS) National High School Questionnaire was utilized (*N* = 16,410) for this study. The YRBS focuses on six major areas of adolescent behaviors: those that lead to intentional and unintentional injuries; tobacco, alcohol and other drug use; sexual behaviors, dietary behaviors; and physical inactivity [3].

The YRBS uses a three-stage cluster sample design to produce a representative sample of 9th through 12th grade students in the US. The sampling frame for the 2009 YRBS consisted of all regular and private schools in at least one of the 9–12 grades in all 50 US states and the District of Columbia (See [33] for a full review). In the first stage, 1,276 primary sampling units (counties, subareas or large counties, or groups of smaller adjacent counties) were grouped into 16 strata based on 1) their metropolitan statistical area, and 2) the percentage of Hispanic and black student represented in the primary sampling units [33]. Fifty-seven primary sampling units were sampled from the 1,276 in 2009.

In the second stage, 196 schools with any grades of 9–12 were sampled with probability proportional to school enrollment size, followed by randomly sampling each of the grades 9–12 in the selected schools in the third stage [33]. A weighting factor was applied to each student record to adjust for non-response and the oversampling of black and Hispanic students in the sample. The final, overall weights were scaled so the weighted count of students was equal to the total sample size, and the weighted proportions of students in each grade matched population projections.

Survey protocols allow for anonymous and voluntary participation to protect the privacy of participating students. Before survey administration, local parental permission procedures were followed. Letters describing the survey were sent to the homes of all targeted participants prior to data collection; parents electing to prevent their child from participating were instructed to return an enclosed form. Trained data collectors who emphasized anonymity, confidentiality, and privacy administered the surveys to the participants in large groups. Students completed the self-administered survey during one class period and recorded their responses directly on a computer-scannable booklet or answer sheet. These procedures were approved by CDC's Institutional Review Board approved for the national YRBS [33].

In 2009, the school response rate was 81% (158 of the 196 sampled schools participated) and the student response rate was 88% (16,460 of the 18,573 sampled students submitted questionnaires). However, 16,410 questionnaires were usable after data editing yielding a 71% overall response rate (81% * 88%) [3].

### 2.2. Measures

The study's independent variable was the YRBS question, “During your life, how many times have you taken a prescription drug (such as OxyContin, Percocet, Vicodin, Adderall, Ritalin, or Xanax) without a doctor's prescription?” Response options for this item were “0 times”, “1–2 times”, “3–9 times”, “10–19 times”, “20 to 39 times”, or “40 or more times”. Age was also included as an independent study variable: “How old are you?” Response options for this question were “12 years old or younger”, “13 years old”, “14 years old”, “15 years old”, “16 years old”, “17 years old”, and “18 years old or older”.

The study's dependent variables were the YRBS questions concerned with suicidal behavior and were “During the past 12 months, did you ever seriously consider attempting suicide? (yes/no)”, and “During the past 12 months, did you make a plan about how you would attempt suicide? (yes/no)”. Two additional questions also served as dependent variables, but had additional response options. For example, the question “During the past 12 months, how many times did you actually attempt suicide?” had “0 times”, “1 time”, “2 or 3 times”, “4 or 5 times”, or “6 or more times” as response options while the question “If you attempted suicide during the past 12 months, did any attempt result in an injury, poisoning, or overdose that had to be treated by a doctor or nurse?” had the response options of “Did not attempt suicide”, “Yes”, or “No”.

The current study's covariates included grade, cigarette smoking, alcohol use, grades, and self-reported feelings of depression. Specifically, self-reported grade was assessed with the question “In what grade are you?” with response options of “9th grade”, “10th grade”, “11th grade”, “12th grade”, and “Ungraded or other grade”. Cigarette smoking was assessed with the question “How old were you when you smoked a whole cigarette for the first time?” with response options of “I have never smoked a whole cigarette”, “8 years older or younger”, “9 or 10 years old”, “11 or 12 years old”, “13 or 14 years old”, “15 or 16 years old”, and “17 years old or older”. Alcohol use was assessed with the question “How old were you when you had your first drink of alcohol other than a few sips?” with the response options of ‘I have never had a drink of alcohol other than a few sips’ with all other response options identical to the cigarette smoking variable. Grade point average was assessed with the question “During the past 12 months, how would you describe your grades in school?” with the response options of “Mostly A's”, “Mostly B's”, “Mostly C's”, “Mostly D's”, “Mostly F's”, “None of these grades”, and “Not sure”. Depression was assessed with the question “During the past 12 months, did you ever feel so sad or hopeless almost every day for two weeks or more in a row that you stopped doing some usual activities? (yes/no)”.

These covariates were chosen because of their consistent independent associations with both NMPDU and suicidal behaviors. For example, increasing age through adolescence, as measured by self-reported grade, has consistently been identified as a correlate to NMPDU [1],[4],[34]–[36] and suicidal behaviors [19],[29]. In addition, both cigarette smoking and alcohol use have been independently associated with illicit drug use [1], NMPDU [13],[37], and suicidal behaviors [38],[52]. Lower grades have also been associated with higher rates of NMPDU [13],[36]. Finally, depression has been found to not only be a predictor of NMPDU [13],[34] and a major risk factor for suicidal behaviors [39],[40],[63], but when combined with substance use is known to have a synergistic influence on suicidal behaviors [25].

### 2.3. Data Analysis

This study sought to determine if NMPDU was related to depression, suicide ideation, plans, and attempts. To investigate this aim, three separate logistic regression models were employed using Proc Surveylogistic in SAS version 9.3 to adjust for the complex YRBS sampling methodology. The first model examined lifetime NMPDU, followed by two additional regressions separated by sex. Models were estimated before and after control for key covariates (age, self-reported grade in school, cigarette smoking, alcohol use, depression, and grade point average).

For these analyses, lifetime NMPDU was dichotomized into those who reported no lifetime NMPDU and compared against those were reported lifetime NMPDU 1+ times in life categories. Since two of the suicidal behavior questions were already dichotomized into “yes” and “no” categories, they did not require any re-coding. However, suicide attempts (last 12 months) was re-coded into those who reported 0 attempts and compared to those who reported 1+ attempts, while the response options of “no” and “did not attempt suicide during the past 12 months” were combined and compared to those who reported requiring medical treatment for their suicide attempt. In all cases, the referent group was the “no use” or “no suicide ideation, or attempts”. None of the covariates were re-coded for analysis.

## 3. Results

### 3.1. Participants

Table 1 details the sample demographic data. The sample was evenly split between males (*N* = 8,065, 49.3%) and females (*N* = 8,280, 50.7%) and grade, but those 14 years old and younger and 18 years or older were underrepresented. As anticipated, fewer students reported fewer suicide attempts (6.4%) and injuries resulting from a suicide attempt (2.0%) than either planning a suicide attempt (11.5%) or seriously considering suicide (14.4%). In addition, approximately 19.8% of the overall sample reported NMPDU in their lifetime (20.6% males, 19.1% females).

**Table 1.** Sample characteristics.

Variable	*n*	%
Sex		
Male	8,065	49.3
Female	8,280	50.7
Age		
≤13 Years	45	0.3
14 Years	1,634	10.0
15 Years	3,701	22.7
16 Years	4,135	25.3
17 Years	4,230	25.9
≥ 18 Years	2,595	15.8

Grade		
9th	4,153	25.4
10th	3,926	24.2
11th	4,092	25.2
12th	4,137	25.2
GPA		
Mostly As	4,162	26.8
Mostly Bs	5,845	38.9
Mostly Cs	3,318	22.2
Mostly Ds	726	4.9
Mostly Fs	311	2.3
None of These	87	0.7
Not Sure	616	4.2
Suicidal Behaviors		
Considered	2,349	14.4
Planned an attempt	1,873	11.5
Attempted	1,053	6.4
Injured by attempt	332	2.0

Rates of self-reported suicidal behavior were also compared between nonmedical prescription drug users and non-users (not tabled) using a series of chi-square analyses. These analyses revealed significant differences between having considered suicide (27.4% users; 11.2% non-users, χ*^2^* = 394.82, *P* < 0.0001), planning a suicide attempt (22.7% users; 8.7% non-users, χ^2^ = 391.74, *P* < 0.0001), suicide attempts (15.0% users; 4.3% non-users, χ^2^ = 406.62, *P* < 0.0001), and an injury needing treatment as a result of a suicide attempt (6.2% users; 1.1% non-users, χ^2^ = 355.15, *P* < 0.0001).

### 3.2. Logistic Regression Findings

Table 2 presents the results from the unadjusted and adjusted regressions. In the unadjusted model, lifetime NMPDU was significantly (*P* < 0.0001) related to all of the suicidal behavior variables with those who reported lifetime NMPDU being between 2.74 and 7.81 times more likely to report suicidal behavior. After covariate adjustment, lifetime NMPDU continued to be significantly (*P* < 0.001) associated with each of the suicidal behaviors with those who reported lifetime NMPDU being between 1.61 and 2.22 times more likely to report suicidal behavior. In both the unadjusted and adjusted models, lifetime NMPDU was most strongly associated with a suicide attempt resulting in an injury that needed treatment.

**Table 2.** Unadjusted and adjusted odds ratios (*OR*) and 95% confidence intervals (*CI*) for reporting suicidal behavior in the past 12 months with covariates included. Nonusers serve as the referent group.

Variable	Unadjusted model*OR* (*CI*)	Adjusted model*OR* (*CI*)
Seriously considered attempting suicide	2.74***(2.39–3.13)	1.61***(1.34–1.94)
Made a plan about attempting suicide	2.90***(2.51–3.35)	1.74***(1.43–2.12)
Attempted suicide	3.77***(3.17–4.49)	1.70***(1.33–2.17)
Suicide attempt resulted in an injury that needed treatment	7.81***(5.64–10.83)	2.22**(1.39–3.54)
Covariates		
Age		0.97(0.89–1.07)
Grade		0.92(0.83–1.01)
GPA		1.08***(1.04–1.13)
Depression		1.46***(1.29–1.65)
Alcohol use		1.54***(1.47–1.60)
Cigarette smoking		1.42***(1.38–1.47)

***P < 0.0001, **P < 0.001.

When the analyses are separated by sex and adjusted for covariates (Table 3), males who reported lifetime NMPDU were 1.61 times more likely to report seriously considering attempting suicide and 1.80 times more likely to report having made a plan to attempt suicide (*P* < 0.01). Lifetime NMPDU continued to be significantly associated with each of the suicide variables for females in the adjusted models. Females who reported lifetime NMPDU were between 1.63 times and 2.32 more likely to report suicidal behavior (*P* < 0.01).

**Table 3.** Adjusted odds ratios (*OR*)† and 95% confidence intervals (*CI*) for reporting suicidal behavior in the past 12 months. nonusers serve as the referent group. Results are provided for females and males separately.

Variable	Females*OR* (*CI*)	Males*OR* (*CI*)
Seriously considered attempting suicide	1.63***(1.29–2.05)	1.62*(1.20–2.20)
Made a plan about attempting suicide	1.74***(1.34–2.26)	1.80**(1.33–2.44)
Attempted suicide	1.82***(1.36–2.46)	1.52(0.97–2.39)
Suicide attempt resulted in an injury that needed treatment	2.32*(1.34–4.03)	2.18(0.87–5.48)

†adjusted for age, self-reported grade in school, grade point average, depression, alcohol use, and cigarette smoking. ***P < 0.0001; **P < 0.001; * *P* < 0.01.

## 4. Discussion

Consistent with a growing body of research [10],[15],[16],[34],[41]–[44], this preliminary study suggests there is a relationship between NMPDU, suicide ideation, plans, and attempts. Furthermore, this study adds significantly to the literature by exploring this relationship among a large, representative sample of adolescents.

Approximately 19.8% of the overall sample reported any NMPDU in their lifetime (20.6% males, 19.1% females). These estimates are consistent with some previous studies examining any NMPDU in adolescent samples (e.g. 20.9% [45]; 22% [46]), lower than results found in recent national samples (24% [5]), and higher than results found in other adolescent samples (10.9% [47]; 10%–13% [48]). These numbers appear to be consistent with results from the 2009 Monitoring the Future (MTF) [4] and the 2009 NSDUH [49], although due to sampling and measurement differences [e.g. definition of NMPDU, time period inquired about (past month, past year, lifetime), year data was collected] a true comparison is difficult to make. The YRBS data generally shows higher prevalence rates than MTF and NSDUH, although long-term trends are similar [1]. Thus, we believe the prevalence rates found in the current study are accurate measures of NMPDU and highlight the significance of NMPDU in this population. Additionally, our age-specific prevalence rates are a particular cause for concern, as adolescents who initiate drug use at younger ages are more likely to struggle with substance abuse than those who initiate at older ages [5].

Findings suggest that after the adjustment for key covariates, including depression, adolescents reporting lifetime NMPDU were significantly more likely to report considering suicide, making a suicide plan, attempting suicide, and injury associated with a suicide attempt. These relationships are important because although the comorbidity of depression and illicit drug use and their association with suicide has been examined in adolescents (e.g. [25],[50],[51]), this relationship has not been examined with prescription drugs. Moreover, the current study makes a unique contribution to the literature by controlling for depression, thus helping to disentangle the complicated issue of comorbidity. Given that those who engage in drug use are at increased odds for suicidal behavior [52] and the high rates of NMPDU in adolescents, study findings emphasize the potential gravity of this relationship.

When separated by sex, males who reported lifetime NMPDU were significantly more likely to report seriously considering attempting suicide and making a suicide plan, while lifetime NMPDU among females continued to be significantly associated with all four suicidal behaviors. These findings are in slight contrast to previous research examining gender differences in suicide behavior(s) in that suicide ideation is typically greater in adolescent females [53], but similar to previous research in that suicide attempts are more likely to occur in adolescent females [54]. The findings, however, are important because not only does suicide ideation predict suicide attempts [55], but both suicide ideation and suicide attempts put adolescents at greater risk for future suicide attempts and completions [53],[55],[56].

Results may also suggest there is something different about females who engage in NMPDU when compared to those who do not, as males typically commit suicide at significantly greater rates than females [53]. Females are more likely to use prescription drugs [1],[56]–[58] and engage in NMPDU for self-treatment [59]. Although speculative, NMPDU may serve as trigger which motivates females to progress from self-treatment use to suicide risk [60].

Although lifetime NMPDU was significantly associated with making a suicide plan for both males and females, the finding for males is a novel finding which we speculate may be indicative of several possible theories. First, males who make a suicide plan may turn to NMPDU because they have no outlet for their psychological distress. Thus, like females they may use prescription drugs to quell their negative affect (e.g. self-medication; see [61]), but unlike females they may be more serious about suicide attempts. Although only 34% of those thinking about suicide develop a suicide plan, approximately 72% of suicidal ideators progress from having a suicide plan to a suicide attempt [62] and those with a plan are over five times more likely to attempt suicide [63]. Second, contrary to the self-medication hypothesis, NMPDU may precede and actually cause or worsen pre-existing psychological issues such as depression and/or suicidal behavior [64] in males. Thus, making a suicide plan may be artifact of NMPDU. Third, NMPDU and suicidal behavior share characteristics such as impulsivity, risk-taking, and sensation seeking. For example, impulse control disorders (e.g. substance use disorders) predict which individuals with suicide ideation go on to make a suicide plan or attempt [65]. And finally, since males tend to choose more lethal methods of suicide such as firearms or hanging [66],[67], perhaps males who make a suicide plan may be under such mental duress that they engage in NMPDU to make the suicide attempt easier. Because the questionnaire asked about lifetime NMPDU, but suicidal behavior in the past year, we are only able to speculate about this finding. Nevertheless, future research should explore this relationship.

Study results also highlight the importance of mental health care and screenings in the adolescent population. For example, recent research suggests that for each year depression was delayed, the odds of lifetime NMPDU decreased by 2.3% [34]. In other words, the earlier the age of major depressive disorder, the greater the odds of lifetime NMPDU. Results do beg the question that if adolescents are treating themselves for psychological distress, why is it that they are not seeking assistance from healthcare providers or other forms of assistance? One explanation may be the lack of access to healthcare often encountered by adolescents. Because adolescents are often under the care and/or coverage of their parents health insurance policy, barriers such as cost, lack of insurance or insurance coverage for certain services, lack of knowledge, and transportation [68], combined with the perceived lack of confidentiality [69],[70], pose a substantial deterrent to seeking and receiving mental health care services. In addition, although school-based health centers are increasingly seen as an entry point to healthcare for adolescents and the vast majority of them do offer some type of mental health services [71], adolescents with depressive disorders are less likely to receive such services than those with disruptive disorders [72]. Stigma and fear of stigma associated with mental illness offer additional barriers to seeking treatment among adolescents [73].

An alternative explanation for the self-treatment of psychological distress may be the ease of access to prescription drugs. The most common sources of prescription drugs are friends or family [74]–[76]. Thus, the barriers associated with seeking treatment may seem insurmountable or excessively arduous when compared to a short walk to the family medicine cabinet or friend's house. In fact, 74% of those borrowing medication from friends or family do so in lieu of seeing a health care provider [77]. Early identification and intervention for treatment of mood disorders may help reduce the severity and/or the persistence of the initial or primary disorder, and prevent theorized related disorders.

Limitations of the current study should be noted. First, we utilized a secondary source of data from the 2009 YRBS Questionnaire. Consequently, our analysis was limited to the questions asked and information collected by this instrument. This may have affected the depressive symptoms and suicidal behavior indicators, as well as the temporal relationship between such variables and NMPDU from these cross-sectional data, including potentially important statistical controls such as stress and/or socioeconomic status. Second, the self-reported nature of the data used in this study could introduce various forms of bias including recall bias, non-response bias, pressure to give desirable answers, and misreporting or inaccurate reporting of variables measured. However, self-reported data on risky behaviors and substance use are generally considered valid [78]. Third, the results cannot be generalized to all adolescents because this sample only included adolescents in grades 9–12 enrolled in public or private schools, and did not include individuals who had dropped out of school or were not present in school on the day of YRBS administration. Fourth, true prevalence of NMPDU and mental health variables measured cannot be determined because questions ask how many times drugs have been used for non-medical reasons over the lifetime and if an individual suffered from mental health issues in the past 12 months. More accurate prevalence estimates would be derived from past 12 month usage. Fifth, because our dependent variable does not distinguish between various types of prescription drugs (e.g., opioid pain killers, stimulants, depressants, sedatives, etc.), it is unknown whether results may be stronger or weaker for any of these differing drug classes. Sixth, we are also unable to identify if other motives were responsible for NMPDU seen in this sample. Despite these limitations, this current study corroborates other studies documenting the prevalence of NMPDU among adolescents, and is among the first to document the association between NMPDU, depression, and suicide ideation, plans, and attempts.

## 5. Conclusion

Controlled medications are important in the treatment of pediatric psychological disorders such as depression and suicidal behavior. Nonetheless, research suggests that an unintended side effect of their greater use and availability may be an increase in NMPDU [46]. Results from the current study suggest that approximately one out of five adolescents have engaged in NMPDU in their lifetime, and that NMPDU is significantly associated with suicidal behavior. Specifically, results suggest that adolescents who report lifetime NMPDU are also at significantly increased odds to engage in suicide ideation even after controlling for key covariates including depression. Moreover, females who reported lifetime NMPDU were also at significantly increased odds to report a suicide attempt and to be injured as a result of their suicide attempt. These results suggest the importance and necessity of continued reinforcement of drug education programs in high school begun in late elementary or middle school. Results also highlight the significance mental health screening and access to healthcare in the adolescent population.
